# Supplementary material for: Identification of photoperiod-induced specific miRNAs in the adrenal glands of Sunite sheep (Ovis aries)
Source: Front Vet Sci. 2022 Jul 22;9:888207. doi: 10.3389/fvets.2022.888207 (PMC9354845; doi:10.3389/fvets.2022.888207)
Supplement: Supplementary file 1 [file Data_Sheet_1.ZIP › Supplementary file/miRDeep2 file.zip!/miRDeep2 file/Novel_Structure/SP42A1/Novel_261.pdf]

Provisional ID	:	NC_019469.2_15447
Score total	:	23.3
Score for star read(s)	:	3.9
Score for read counts	:	15.9
Score for mfe	:	1.8
Score for randfold	:	1.6
Score for cons. seed	:	
Total read count	:	43
Mature read count	:	19
Loop read count	:	0
Star read count	:	24

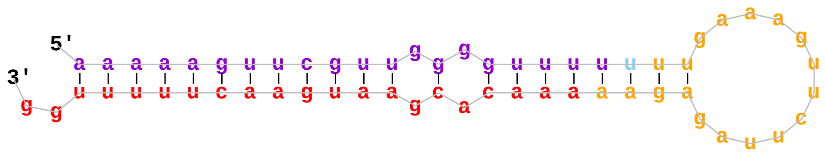

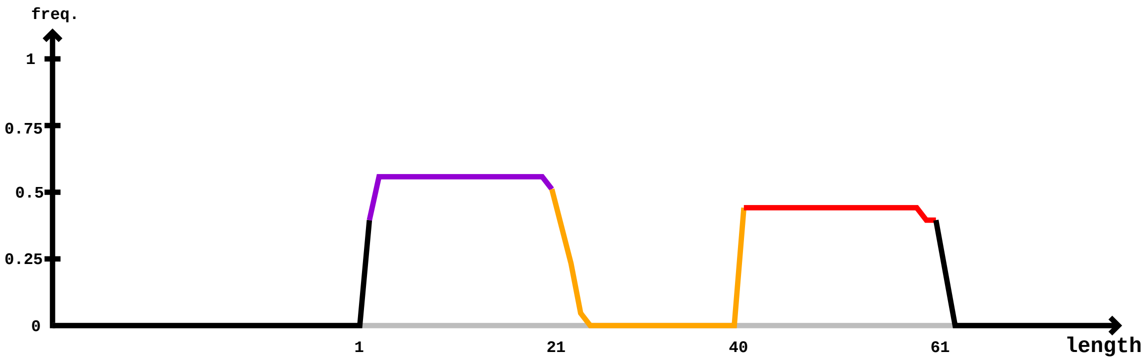


```
                                              Star                                    Mature
5'- gcuuggguggcaccaaaauauugggauggccaaaaaguucguugggguuuuuuugaaaguucuuagagaaaaacacgaaugaacuuuuuggcuaguccuaaagaccccu  -3'  obs
    gcuuggguggcaccaaaauauugggauggccaaaaaguucguugggguuuuuuugaaaguucuuagagaaaaacacgaaugaacuuuuuggcuaguccuaaagaccccu       exp
    ..((((......))))....((((((((((((((((((((((.(.((((((((...........)))))))).).))))))))))))))))).))))))........  reads   mm   sample
    ...............................aaaaaguucguuUggguuu...........................................................  2       1    seq
    ...............................aaaaaguucguuUggguuuu..........................................................  12      1    seq
    ...............................aaaaaguucguuUggguuuuuu........................................................  3       1    seq
    ................................aaaaguucguuUggguuuuuu........................................................  5       1    seq
    ................................aaaaguucguuUggguuuuuuu.......................................................  2       1    seq
    ......................................................................aaacacgaaugaacuuuuu....................  1       0    seq
    ......................................................................aaacUcgaaugaacuuuuu....................  1       1    seq
    ......................................................................aaacCcgaaugaacuuuuugg..................  17      1    seq
```
